# Sesamol counteracts on metabolic disorders of middle-aged alimentary obese mice through regulating skeletal muscle glucose and lipid metabolism

**DOI:** 10.29219/fnr.v66.8231

**Published:** 2022-03-17

**Authors:** Min-Min Hu, Ji-Hua Chen, Quan-Quan Zhang, Zi-Yu Song, Horia Shaukat, Hong Qin

**Affiliations:** Department of Nutrition Science and Food Hygiene, Xiangya School of Public Health, Central South University, Changsha, China

**Keywords:** sesamol, obesity, aging, skeletal muscle, glucose metabolism, lipid metabolism

## Abstract

**Background:**

Globally, obesity is a significant public problem, especially when aging. Sesamol, a phenolic lignan present in sesame seeds, might have a positive effect on high-fat diet (HFD)-induced obesity associated with aging.

**Objective:**

The purpose of current research study was to explore salutary effects and mechanisms of sesamol in treating alimentary obesity and associated metabolic syndrome in middle-aged mice.

**Methods:**

C57BL/6J mice aged 4–6 weeks and 6–8 months were assigned to the young normal diet group, middle-aged normal diet group, middle-aged HFD group, and middle-aged HFD + sesamol group. At the end of experiment, glucose tolerance test and insulin tolerance test were performed; the levels of lipids and oxidative stress-related factors in the serum and skeletal muscle were detected using chemistry reagent kits; lipid accumulation in skeletal muscle was observed by oil red O staining; the expressions of muscular glucose and lipid metabolism associated proteins were measured by Western blotting.

**Results:**

Sesamol decreased the body weight and alleviated obesity-associated metabolism syndrome in middle-aged mice, such as glucose intolerance, insulin resistance, dyslipidemia, and oxidative stress. Moreover, muscular metabolic disorders were attenuated after treatment with sesamol. It increased the expression of glucose transporter type-4 and down-regulated the protein levels of pyruvate dehydrogenase kinase isozyme 4, implying the increase of glucose uptake and oxidation. Meanwhile, sesamol decreased the expression of sterol regulatory element binding protein 1c and up-regulated the phosphorylation of hormone-sensitive lipase and the level of carnitine palmityl transferase 1α, which led to the declined lipogenesis and the increased lipolysis and lipid oxidation. In addition, the SIRT1/AMPK signaling pathway was triggered by sesamol, from which it is understood how sesamol enhances glucose and lipid metabolism.

**Conclusions:**

Sesamol counteracts on metabolic disorders of middle-aged alimentary obese mice through regulating skeletal muscle glucose and lipid metabolism, which might be associated with the stimulation of the SIRT1/AMPK pathway.

## Popular scientific summary

- Sesamol attenuates HFD-induced obesity, its related metabolic syndrome and muscular metabolic disorders in the middle-aged mice, which might result in the regulation of glucose and lipid metabolism in the skeletal muscle.
- Skeletal muscle is crucial for the utilization of glucose and lipid. Sesamol can decrease anabolic energy-consuming pathways (lipogenesis) and increase catabolic energy-producing pathways (glucose oxidation, lipolysis, and fatty acid oxidation), which might be associated with the stimulation of the SIRT1/AMPK pathway.

Obesity is mainly caused by energy imbalance, which has been linked to glucose and lipid metabolic disorders. These pathological conditions have resulted in the early onset of many chronic diseases (1, 2), particularly when aging, which has produced a significant challenge to the global public health (3, 4).

Skeletal muscle is regarded as the key metabolic organ for the regulation of systemic energy homeostasis, and it is important for the utilization of glucose and free fatty acids (5–7). Present-day data showed that imbalance of the glucose and lipid metabolism in the skeletal muscle was associated with obesity, especially in the context of aging, were in due course leading to metabolic syndrome (8–10). Metabolic syndrome represents a constellation of metabolic disturbances, such as glucose intolerance, insulin resistance, dyslipidemia, and elevated oxidative stress (11). Therefore, therapeutic strategies that could reduce the obesity-induced glucose and lipid metabolic disorders in skeletal muscle might be considered as a crucial factor for rectifying metabolic syndrome in obese individuals during the aging process.

Phytochemicals have shown positive effects in treating various diseases, including obesity and related metabolic syndrome (12, 13). Sesamol (5-hydroxy-1,3-benzodioxole), a water-soluble phenolic lignan present in sesame seeds, exhibited miscellaneous biological activities (14, 15). Our previous data had suggested that sesamol could relieve alimentary obesity and obesity-related hepatic glucose and lipid metabolic disorders in young adult mice (16, 17). However, the therapeutic effects of sesamol in alimentary obesity associated with aging, as well as its underlying mechanisms, remain unclear. In this research study, we aimed to investigate whether sesamol could attenuate obesity and its related metabolic syndrome via modulating lipid and glucose metabolism of skeletal muscle in the middle-aged mice.

## Materials and methods

### Materials

C57BL/6J male mice (4–6 weeks and 6–8 months of age) were obtained from Central South University (Changsha, China). Sesamol was purchased from Sigma-Aldrich (St. Louis, MO). An insulin assay kit was obtained from Cusabio (Wuhan, China). Triglyceride (TG, A110-1-1), total cholesterol (TC, A111-1-1), low-density lipoprotein cholesterol (LDL-C, A113-1-1), high-density lipoprotein cholesterol (HDL-C, A112-1-1), malondialdehyde (MDA, A003-1-2), and superoxide dismutase (SOD, A001-1-2) assay kits were acquired from the Institute of Jiancheng Bioengineering (Nanjing, China). Oil Red O staining solution was obtained from Beijing Solarbio Science & Technology Co. Ltd (Beijing, China). Protease inhibitor was provided by Dingguo Changsheng Biotechnology Co. Ltd (Beijing, China). The bicinchoninic acid (BCA) protein assay kit was obtained from Beyotime Biotechnology, Inc. (Shanghai, China). Antibodies against glucose transporter protein type-4 (GLUT4, A7637), pyruvate dehydrogenase kinase isozyme 4 (PDK4, A13337), sterol regulatory element-binding protein 1 c (SREBP-1c, A15586), carnitine palmityl transferase 1α (CPT1α, A5307), AMP-activated protein kinasea1/a2 (AMPK, A12718), phospho-AMPKa1-T183/AMPKa2-T172 (p-AMPK, AP0116), sirtuin 1 (SIRT1, A19667), α-tubulin (AC007), and horseradish peroxidase-conjugated secondary antibody (AS014) were obtained from ABclonal, Inc. (Boston, USA). Antibodies against glycogen synthase (GS, ab40810) and phosphor-GS (p-GS, ab81230) were obtained from Abcam (Cambridge, UK). Antibodies against hormone-sensitive lipase (HSL, AF6403) and phospho-HSL (p-HSL, AF8026) were obtained from Affinity (Cincinnati, OH, USA).

### Animals and diet

The mice were kept under control conditions of optimum temperature (20–24°C) with 12-h light /12-h dark cycle. Animals had accessibility to food and tap water ad libitum. First, the mice were randomized into three groups: 1) the mice aged 4–6 weeks (*n* = 7) assigned to the youth ND group (YND) were fed with the normal diet (ND) (rodent diet D12450B, Research Diets Inc., USA), 2) the mice aged 6–8 months (*n* = 7) assigned to the middle-aged ND group (MND) were fed with ND, 3) the mice aged 6–8 months (*n* = 21) assigned to the middle-aged HFD group to establish the obese model that were fed with the high-fat diet (HFD) (rodent diet D12492, Research Diets Inc., USA). After 8 weeks, middle-aged HFD-fed mice with 20% more weight when compared with MND-fed mice were designated as obese, the bulkiest 14 mice were carefully chosen for the succeeding experiment. Then, the 14 middle-aged HFD-fed mice were categorized into two weight-matched groups: the middle-aged HFD group (MHFD, *n* = 7) and the middle-aged HFD + sesamol group (MHFD + SEM, *n* = 7). HFD or ND diet was provided to all the mice for 8 more weeks. Sesamol was dissolved in 0.5% carboxymethyl cellulose and administered once daily (100 mg/kg body weight). Mice in other groups were gavaged with the same volume of vehicle only (0.5% carboxymethyl cellulose). Food was changed and measured daily. Food intake was acquired by subtracting the remaining food from the amount of original food. Energy intake was calculated in accordance with 3.85 kcal/g for the ND (10 kcal% fat, 70 kcal% carbohydrate, 20 kcal% protein) and 5.24 kcal/g for the HFD (60 kcal% fat, 20 kcal% carbohydrate, 20 kcal% protein). The ethical treatment was given to animals in this research. All the experiments used in this research study were approved by the Institutional Animal Care and Use Committee of Central South University (XYGW-2020-34).

### Intraperitoneal glucose tolerance test, intraperitoneal insulin tolerance test, fasting blood glucose, serum insulin and homeostasis model assessment of insulin resistance (HOMA-IR)

On the 16th week, intraperitoneal glucose tolerance test (IPGTT) and intraperitoneal insulin tolerance test (IPITT) were conducted. According to previous studies (16, 17), for IPGTT, the animals were fasted for 12 h, followed by intraperitoneal injection of glucose at 2 g/kg body weight. Blood glucose levels were monitored from tail vein blood before and after the glucose injection (at 0, 15, 30, 60, and 120 min) using a blood glucose meter (Contour TS, Bayer, Germany); for IPITT, the animals were fasted for 12 h. After basal glycemic measurement, the serum glucose concentrations were determined from tail vein at 15, 30, 60, and 120 min after intraperitoneal injection of 0.75 unit insulin/kg body weight. The levels of fasting blood glucose (FBG) and serum insulin were examined from the tail lip in fasted mice. The following equation was used to calculate HOMA-IR: FBG concentration (mmol/L) × fasting insulin concentration (mU/L) / 22.5.

### Serum and tissue collection

After 16 weeks, serum samples were drawn from the femoral artery and prepared by centrifugation. Afterwards, the serum was stored at −80°C until analysis. Subsequently, mice were executed for tissue collection. Gastrocnemius (GAS) muscle tissues were either immediately fixed in 4% formalin solution or quickly frozen in liquid nitrogen.

### Serum biochemical analyses

The contents of TG, TC, LDL-C, HDL-C and MDA, and the activity of SOD were measured using chemistry reagent kits, as described in the manufacturer’s instruction.

### Skeletal muscle biochemical analyses

The skeletal muscle samples were homogenized with physiological saline, followed by centrifugation (590× *g* for 10 min at 4°C). The levels of MDA and TG, and the activity of SOD in skeletal muscle were determined using chemistry reagent kits.

### Lipid accumulation analysis in skeletal muscle

Lipid accumulation in the skeletal muscle tissues were examined by oil red O staining. Fixed GAS muscle tissues were dehydrated by 4% formalin solution, then paraffin-embedded, cut into section, and stained with oil red O staining solution. Afterwards, these sections were viewed under an optical microscope (EVOSTM Auto2, Thermo Fisher Scientific, WA, USA). The area of red lipid droplet and GAS were measured by Image Pro Plus software, and the area of red lipid droplet was normalized by GAS area.

### Western blot analysis

GAS muscle tissues were lysed in cold lysis buffer containing protease inhibitor. Next, these homogenates were centrifuged (13,680*g* for 15 min at 4°C) for supernatant collection. The protein contents were quantified by BCA protein assay kit. These proteins were divided by sodium dodecyl sulfate polyacrylamide gel electrophoresis (SDS-PAGE) under reducing conditions and transmitted to polyvinylidene fluoride membranes. Membranes were incubated for 1 h at room temperature in 5% non-fat milk, then washed and immunoblotted overnight on a rocking platform at 4°C with primary antibodies: GS, P-GS, GLUT4, PDK4, SREBP1c, HSL, p-HSL, CPT1α, SIRT1, AMPK, p-AMPK, and α-tubulin. After washing, these membranes were incubated in the secondary antibody for 1 h. The bands on membranes were visualized using the chemiluminescence imager (Tanon-5500, Tanon Science & Technology Co., Ltd, Shanghai, China) with ECL. The target band densities were analyzed using Image J software.

### Statistical analysis

Statistical analysis was carried out by the SPSS 20.0 statistical program. Data were expressed as mean ± standard deviations (SD). Comparison among the groups was made via one-way analysis of variance (ANOVA). A least-significant difference t-test was used to determine whether there is a significant difference between the parameters. *P* < 0.05 was considered to be statistically significant.

## Results

### Sesamol alleviated alimentary obesity in middle-aged mice

**Fig. 1:**
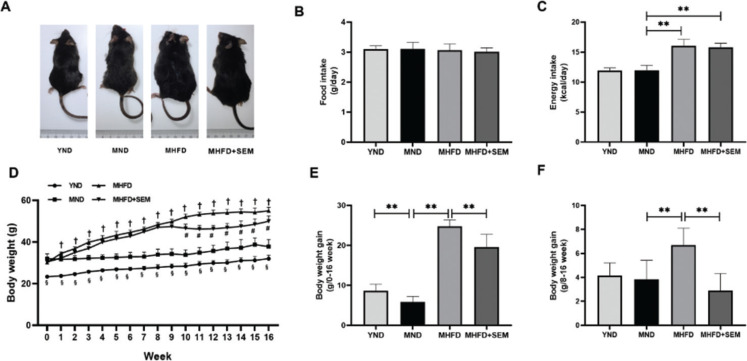
Effects of sesamol administration on the body weight in the middle-aged mice. (a) Representative images at 16 weeks. (b) Food intake (g/day). (c) Energy intake (kcal/day). (d) Body weight changes with time. (e) Body weight gain from 0 to 16 week. (f) Body weight gain from 8 to 16 week. Results are represented as means ± SD (*n* = 7 per group). *P < 0.05, **P < 0.01. In Fig. d, ^§^, ^†^, ^#^ represent statistically significant differences between the YND group and the MND group, the MND group and the MHFD group, the MHFD group and the MHFD+SEM group, respectively.

The body weight gain was remarkably higher in the YND group than in the MND group, (Fig. 1d, f), because at that time young mice were going through the critical period of its growth and development (18). In middle-aged mice, there were no significant differences in food take (Fig. 1b), indicating that HFD and sesamol did not affect the appetite of mice. Compared with the MND group, the MHFD and MHFD + SEM group had a higher energy intake (Fig. 1c). Moreover, the MHFD group gained more body weight than the MND group, while the MHFD+SEM group displayed a vigorous reduction in body weight gain compared with the MHFD group (Fig. 1d–f). These results suggested that sesamol treatment had diminished the problem of excessive weight gain caused by HFD of the middle-aged mice.

### Sesamol attenuated insulin resistance in middle-aged obese mice

**Fig. 2:**
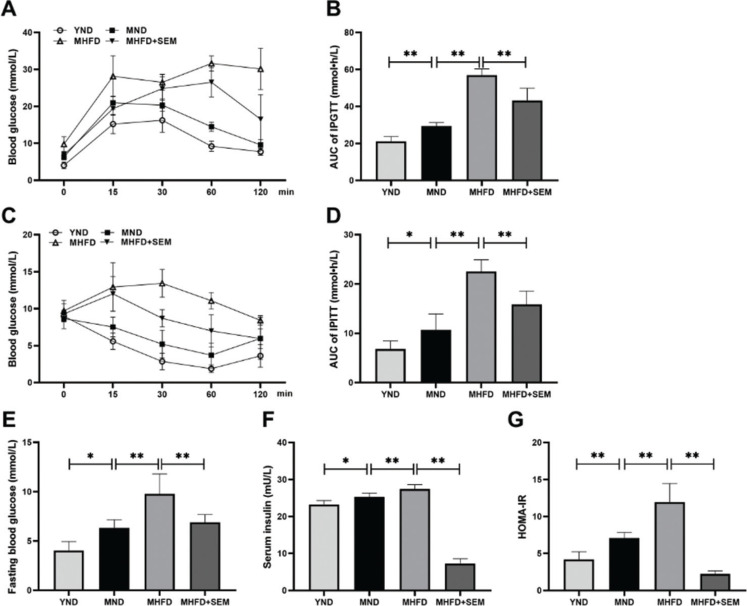
Effects of sesamol intervention on insulin sensitivity in the middle-aged mice. (a) Blood glucose concentrations at 0, 15, 30, 60, and 120 min after glucose injection intraperitoneally at 16 weeks. (b) Area under the curve (AUC) of IPGTT. (c) Blood glucose levels at 0, 15, 30, 60, and 120 min after insulin injection intraperitoneally at 16 weeks. (d) AUC of IPITT. (e) Glycemia after 12 h fasting. (f) Serum insulin concentrations after 12 h fasting. (g) HOMA-IR. Results are represented as means ± SD (*n* = 5 per group). *P < 0.05, **P < 0.01.

To explore the effects of sesamol on obesity-related insulin resistance, the IPGTT and IPITT were performed. The MND group showed higher area under the curve (AUC) of IPGTT and IPITT, FBG, fasting insulin and HOMA-IR than the YND group (Fig. 2a–e). These data indicated that the problem of aging led to impaired glucose tolerance and insulin sensitivity. Moreover, the serum levels of FBG, fasting insulin, HOMA-IR, and the AUC of IPGTT and IPITT in HFD-fed middle-aged mice manifested observable increase, when compared with ND-fed middle-aged mice, which were significantly reduced after sesamol intervention. Hence, we concluded that sesamol could attenuate insulin resistance and glucose intolerance in the middle-aged HFD-fed mice.

### Sesamol ameliorated dyslipidemia in middle-aged obese mice

**Fig. 3:**
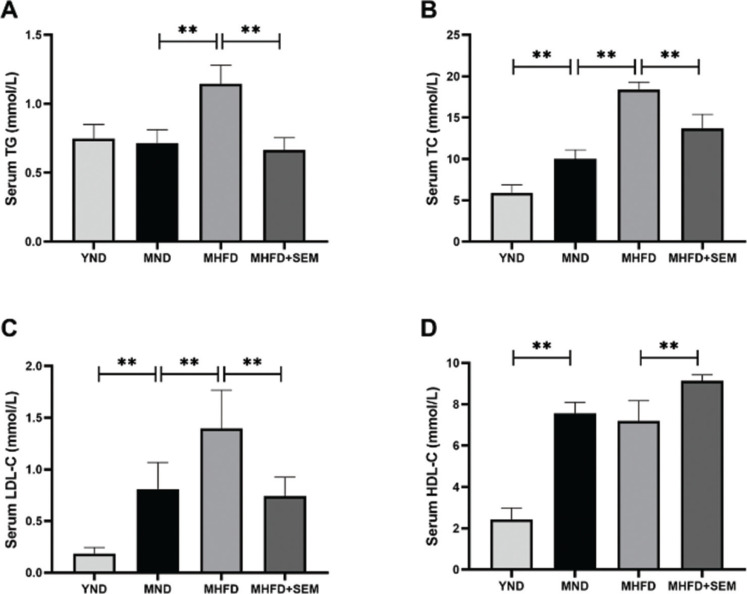
Effects of sesamol administration on the serum lipid profile in the middle-aged mice. (a) Serum TG. (b) Serum TC. (c) Serum LDL-C. (d) Serum HDL-C. Results are represented as means ± SD (*n* = 5 per group). *P < 0.05, **P < 0.01.

To evaluate whether sesamol intervention could relieve obesity-associated dyslipidemia, we measured the serum levels of TG, TC, LDL-C and HDL-C. The MND group exhibited appreciably higher serum levels of TC, LDL-C and HDL-C than the YND group (Fig. 3b–d), meaning that the levels of serum lipid increased with age. In addition, the serum levels of TG, TC and LDL-C in the MHFD group were markedly higher than those in the MND group, while sesamol treatment significantly reduced the serum concentrations of TG, TC, LDL-C and enhanced the serum concentration of HDL-C (Fig. 3a). The data demonstrated that sesamol attenuated obesity-related dyslipidemia in middle-aged obese mice.

### Sesamol alleviated oxidative stress in middle-aged obese mice

**Fig. 4:**
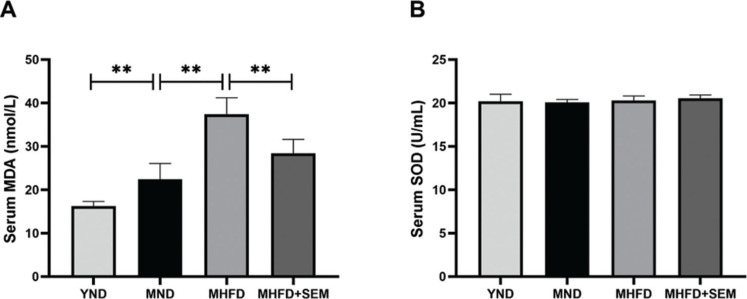
Effects of sesamol intervention on oxidative stress in the middle-aged mice. (a) Serum MDA. (b) Serum SOD. Results are represented as means ± SD (*n* = 5 per group). *P < 0.05, **P < 0.01.

To assess whether sesamol could attenuate obesity-associated oxidative stress, the level of serum MDA and the activity of serum SOD were evaluated. Our data showed that serum MDA levels were remarkably increased in ND-fed middle-aged mice compared with HFD-fed middle-aged mice, indicating that aging elevated oxidative damage (Fig. 4a). Meanwhile, compared with the MND group, the MHFD group had considerably a higher serum MDA concentration, and sesamol markedly diminished the level of serum MDA. The activity of serum SOD indicated no significant differences among the groups (Fig. 4b). Our study results revealed that sesamol could reduce oxidative stress in middle-aged obese mice.

### Sesamol partially attenuated muscular metabolic disorders in middle-aged obese mice

**Fig. 5:**
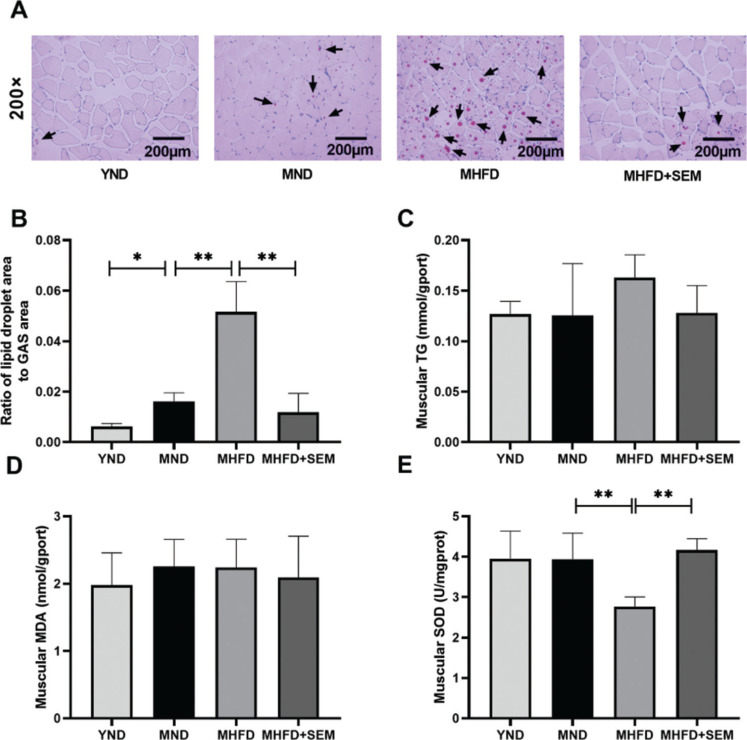
Effects of sesamol administration on muscular metabolic disorders in the middle-aged mice. (a) Representative images of Oil Red O staining (200×), scale bar = 200 μm. Arrow heads represent lipid droplets. (b) Ratio of lipid droplet area to GAS area. (c) Muscular TG. (d) Muscular MDA. (e) Muscular SOD. Results are represented as means ± SD (*n* = 5 per group). *P < 0.05, **P < 0.01.

After providing the close association between metabolic syndrome and muscular metabolic disorders, we further sought to evaluate whether sesamol could alleviate skeletal muscle metabolic disorders. First, we evaluated the muscular lipid accumulation in the middle-aged obese mice. When compared with the YND group, the MND group showed a remarkably increased relative area of lipid droplet (Fig. 5a, b). Meanwhile, the ratio of lipid droplet area to GAS area was adequately increased in the MHFD group when compared with the MND group, while the sesamol-treated group had a smaller relative area of lipid droplet than the MHFD group. In terms of the muscular TG level, there were no significant differences between groups (Fig. 5c). These findings revealed that sesamol could partially reduce the level of lipid accumulation in middle-aged HFD-fed mice.

Furthermore, we determine the effects of sesamol on middle-aged obesity-related muscular oxidative stress. The muscular SOD activity of the MHFD group was remarkably lower than that of the MND group and also than the sesamol-administered groups (Fig. 5e). The muscular MDA level did not vary between groups (Fig. 5d). These data suggested that the capacity of antioxidant enzyme in the skeletal muscle was increased by sesamol treatment in middle-aged mice. Altogether, sesamol might reverse the muscular metabolism disorders in the middle-aged obese mice, including lipid accumulation, and partially enhanced the capacity of antioxidant enzyme.

### Sesamol regulated muscular lipid and glucose metabolism in middle-aged obese mice

**Fig. 6:**
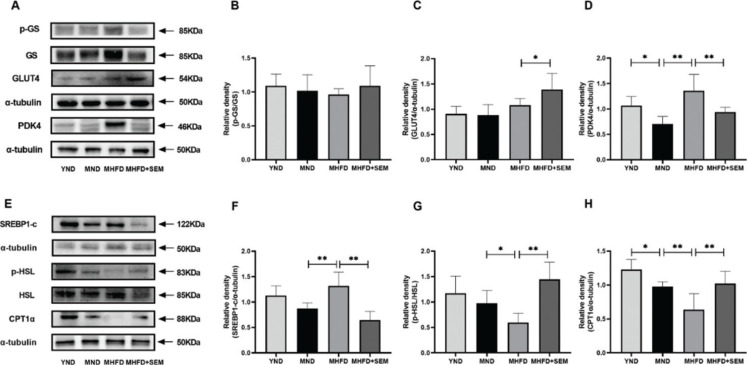
Effects of sesamol intervention on the expressions of glucose and lipid metabolism-related proteins in skeletal muscle of the middle-aged mice. (a) Representative western blot images and quantification of (b) p-GS and GS, (c) GLUT4 and (d) PDK4. (e) Representative western blot images and quantification of (f) SREBP1-c, (g) p-HSL and HSL and (h) CPT1α. *P < 0.05, **P < 0.01.

To investigate the potential mechanism that sesamol ameliorate the metabolic syndrome and the muscular metabolic disorders in the middle-aged obese mice, we detected the levels of several representative glucose and lipid metabolism-related proteins in the skeletal muscle. The levels of glucose and lipid metabolism-related proteins were down-regulated in the MND group, and the levels of PDK4 and CPT1α were prominently decreased when compared with the YND group. The above results indicated that as the age increases, the levels of glucose and lipid metabolism-related proteins were partially down-regulated. Furthermore, there were no differences in the expressions of p-GS among the four groups, which suggested that sesamol does not affect glycogen synthesis in skeletal muscle (Fig. 6a, b). However, the level of GLUT4 was increased by sesamol treatment, suggesting that sesamol could elevate glucose uptake (Fig. 6c). Meanwhile, compared with the MND group, the expression of PDK4 was remarkable increased in the MHFD group, whereas sesamol intervention had decreased the expression of PDK4 (Fig. 6d), which indicated that sesamol could increase the glucose oxidation in the skeletal muscle. However, the expression of SREBP1-c for lipogenesis was diminished by sesamol treatment (Fig. 6e, f). Moreover, the phosphorylation of lipolytic HSL protein and the level of CPT1α protein involved in fatty acid β-oxidation were significantly increased after sesamol intervention (Fig. 6 g, h). These results revealed that sesamol markedly reduced the process of lipogenesis, and increased the process of lipolysis and lipid β-oxidation in the skeletal muscle. Overall, the data revealed that sesamol regulated glucose and lipid metabolism in the skeletal muscle of middle-aged obese mice.

### Sesamol regulated glucose and lipid homeostasis via activating muscular SIR1/AMPK pathway in middle-aged obese mice

**Fig. 7:**
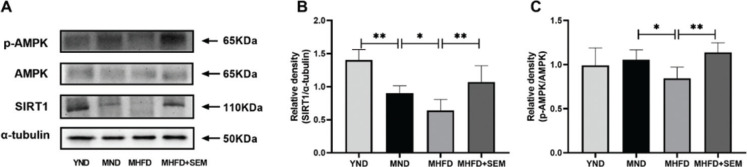
Effects of sesamol administration on expressions of SIRT1/AMPK pathway in skeletal muscle of the middle-aged mice. (a) Representative western blot images and quantification of (b) SIRT1, and (c) p-AMPK and AMPK. Results are represented as means ± SD (*n* = 5 per group). *P < 0.05, **P < 0.01.

AMPK has been reported to be an upstream kinase of SREBP1-c, HSL, CPT1α, GLUT4, and PDK4, and it is one of the key regulating factors of the skeletal muscle energy metabolism (19–21). Furthermore, SIRT1 is involved in AMPK activation, and it is also a critical regulator for energy metabolism (21). To further illuminate the molecular mechanism that sesamol exerted on its metabolic regulatory effects, we examined the p-AMPK/AMPK ratio and the expression of SIRT1 in mice. The expressions of SIRT1 in the MND group were declined adequately when compared with the YND group (Fig. 7a, b). In addition, the level of SIRT1 protein was significantly increased after sesamol administration. Meanwhile, sesamol intervention notably enhanced the phosphorylation of AMPK (Fig. 7c), which demonstrated that sesamol might improve glucose and lipid metabolism via activating the muscular SIRT1/AMPK pathway in the middle-aged obese mice.

## Discussion

In the current research, we explored the novel efficacy and potential mechanisms of sesamol in the treatment of alimentary obesity associated with aging. We found out that the body weight gain was decreased and obesity-related metabolic syndrome was ameliorated in middle-aged mice by sesamol treatment. These salutary effects were correlated with the stimulation of SIRT1/AMPK pathway in the skeletal muscle.

Consistent with the previous findings, our results indicated that middle-aged mice had suffered more from metabolic disorders when compared with the young mice (22, 23), which reminded us that the health condition deteriorates with age, and hence, needed widespread attention. Moreover, aged individuals with western diet, high in fat, have an increased risk of obesity-related metabolic syndrome, including glucose intolerance, insulin resistance, dyslipidemia, and elevated oxidative stress (24, 25), which are the major risk factors of noncommunicable chronic diseases (26). Previous studies had revealed that sesamol had the ability to alleviate metabolic parameters that were related to obesity in young adult mice (17, 27, 28); however, the effects of this compound on obesity associated with aging were not examined. In the current study, we provided the evidence that sesamol could attenuate obesity-related metabolic syndrome in the middle-aged mice. First, 100 mg/kg body weight sesamol administration significantly lessened the body weight gain in the HFD-fed middle-aged mice. In addition, we found out that sesamol improved glucose disposal and insulin sensitivity, thus, middle-aged obese mice had lower AUC of IPGTT and IPITT, levels of FBG and HOMA-IR after sesamol intervention. Furthermore, sesamol improved the serum lipid profile, as the serum levels of TG, TC, and LDL-C were considerably reduced and the serum HDL-C concentration was increased by sesamol treatment. Sesamol had also notably decreased the oxidative stress in the middle-aged HFD-fed mice, and hence, presented a decline in the serum concentration of MDA. As the main product of lipid peroxidation, the depletion of MDA level means the reduction of oxidative stress. Collectively, these findings indicated that sesamol showed positive effects in treating obesity and its associated metabolic syndrome of middle-aged mice.

Skeletal muscle is well known as one of the fundamental tissues in regulating energy metabolism (29, 30). Obesity-related high-fat condition consistently causes metabolic disturbances in the skeletal muscle, which, in turn, aggravates systemic metabolic syndrome, especially when aging (31–33). Considering that ameliorating muscular metabolic abnormalities is one of the effective therapeutic approaches in treating obesity and its related metabolic syndrome (34), we speculated that sesamol alleviated metabolic syndrome under obesity condition by regulating metabolism in the skeletal muscle. The results indicated that sesamol ameliorated obesity-induced muscular metabolic disorders in middle-aged mice, such as lipid accumulation and oxidative stress. Obesity is characterized by increase in body fat mass, which could cause ectopic lipid accumulation in muscles (35). Herein, we discovered that sesamol significantly reduced skeletal muscle lipid droplets in middle-aged obese mice. Moreover, oxidative stress, one of the typical muscular metabolic abnormalities, was elevated under the condition of obesity (36). SOD is one of the key enzymatic antioxidants, which plays a pivotal role in reducing oxidative stress (37). Our data showed that muscular activity of SOD was enhanced after sesamol intervention, which suggested that sesamol increased the capacity of antioxidant enzyme in the skeletal muscle. Considering that sesamol could decrease the serum MDA level, we assumed that sesamol might elevate the activity of antioxidant enzyme in the skeletal muscle to relieve systemic oxidative stress. In this study, however, no change in the muscular MDA content was observed, which might be attributed to the fact that skeletal muscle is not the main site where the oxidative stress occurs. These findings demonstrated that sesamol treatment could partially attenuate skeletal muscle metabolic disorders to alleviate the obesity-related metabolic syndrome in the middle-aged mice.

Afterwards, we focused on the molecular mechanism that sesamol ameliorating muscular metabolic disturbances in the middle-aged mice. Muscular dysregulated glucose and lipid metabolism is the hallmark of the metabolic anomalies in obesity populations (38). Hence, the inhibition of anabolism and the stimulation of catabolism of glucose and lipid are regarded as an achievable method to restrain from these abnormalities. First, we evaluated the effects of sesamol on muscular glucose metabolism in the middle-aged obese mice. It is commonly agreed that GS is a vital enzyme in regulating glycogen biosynthesis that is inactivated after phosphorylation (39). Our results revealed that the phosphorylation level of GS was unaffected by sesamol treatment, indicating that sesamol might not regulate glycogen biosynthesis. Then, the effect of sesamol on glucose uptake was detected. When stimulated by insulin, GLUT4 can facilitate glucose uptake in the skeletal muscle (40), the expression of GLUT4 was elevated by sesamol treatment, meaning that sesamol can increase glucose uptake. Moreover, we determined the effects of sesamol on glucose oxidation. PDK4 is one of the essential rate-controlling enzymes for glucose oxidation, which can phosphorylate pyruvate dehydrogenase (PDH) to suppress the catalysis of pyruvate to acetyl-CoA, resulting in reduced glucose oxidation (41). In line with the former studies that PDK4 expression levels were increased in the obese state (42), the level of PDK4 in this study was enhanced in the obese model; however, it was noticeably down-regulated after delivering sesamol. The above-mentioned verifications indicated that sesamol intervention had a beneficial impact on muscular glucose metabolism by up-regulating the glucose uptake and oxidation in the middle-aged obese mice. Next, we examined the effects of sesamol on lipid metabolism, which were mainly determined through lipogenesis and lipid catabolism, including lipolysis and the oxidation of fatty acid. It is generally accepted that cholesterol and lipogenesis are modulated by SREBP1-c, because SREBP1-c can stimulate the transcription of several lipogenic genes, including fatty acid synthase and stearoyl-CoA desaturase 1 (43). We also noted that there was a definite decline in the muscular SREBP1-c protein expression after sesamol intervention, thereby indicating the repression of the lipid synthesis. In terms of lipolysis, HSL is one of the important lipases that control the rate of lipolysis and can be activated by phosphorylation (44). We found out that sesamol considerably uplifted the phosphorylation level of HSL in the skeletal muscle tissue, which suggested that sesamol could increase muscular lipolysis in the middle-aged obese mice. However, the enhancement of lipolysis may result in an increase in the fatty acid, which, in turn, enters the blood circulation (45), further disturbing the muscular function and insulin signaling and eventually leading to the problem of insulin resistance (46). In the current study, sesamol increased lipolysis in the skeletal muscle, but as a result, insulin resistance was alleviated. Thus, we hypothesized that sesamol could up-regulate fatty acid oxidation simultaneously. CPT1α is considered as a primary rate-limiting enzyme, which promotes the diversion of long-chain acyl-CoA to mitochondria for β-oxidation (47). The results were consistent with our predictions that orally administered sesamol significantly up-regulated the content of muscular CPT1α protein in the middle-aged obese mice, demonstrating that lipid oxidation was enhanced by sesamol. Collectively, we concluded that sesamol alleviated skeletal muscle metabolic abnormalities by increasing anabolism and decreasing catabolism of glucose and lipid in the middle-aged obese mice.

AMPK, a crucial cellular energy sensor, is responsible for maintaining glucose and lipid homeostasis in the skeletal muscle (48). Muscular decreased AMPK activity has been detected in the obese animals and it has a close connection with the development of metabolic disturbances (49, 50). While in reverse, specifically activating this protein contributes to the attenuation of obesity-associated muscular metabolic disorders (51). Moreover, after activated by phosphorylation, AMPK can reduce the expression of PDK4 and SREBP1-c, elevate the phosphorylation of HSL and the expression of GLUT4 and CPT1α (19–21), and thus, eventually leading towards the amelioration of anabolic energy-consuming pathways (lipogenesis) and the activation of catabolic energy-producing pathways (glucose oxidation, lipolysis, and fatty acid oxidation) to restore energy homeostasis in cells (52). Therefore, it is possible that AMPK is a target of sesamol. As expected, we found out that sesamol elevated the content of phosphorylated AMPK, suggesting that sesamol could activate AMPK to control the metabolism of lipid and glucose in skeletal muscle of the middle-aged obese mice.

Furthermore, previous evidence demonstrated that SIRT1 is of critical importance in AMPK activation, and it is also a pivotal protein in modulating glucose and lipid metabolism (21). We assumed that SIRT1 was the other potential target of sesamol. So, the SIRT1 content was detected in the skeletal muscle of mice. Similar to previous studies, in both aging and obesity, the level of SIRT1 was abnormally reduced in skeletal muscle (53). In addition, after being treated with sesamol, there was a remarkable increase in the SIRT1 content. As a NAD+-dependent histone deacetylases, SIRT1 can deacetylate specific enzymes and transcription factors to directly or indirectly influence the activities of downstream substrates, including AMPK to maintain the homeostasis of glucose and lipid (54). Overall, these results indicated that sesamol could activate the muscular SIRT1/AMPK signaling pathway, and it might be the underlying mechanism for the regulation of muscular glucose and lipid metabolism in the middle-aged obese mice.

Besides the glucose and lipid metabolism, one must consider the anti-obesity impact of sesamol by enhancing energy consumption and inhibiting nutrient utilization, etc. In further research, we will measure the animal habitual activity, VO_2_, and energy content of the animal feces to comprehensively understand the effects of sesamol on obesity and aging-related metabolic disorders. Meanwhile, although the animal testing could provide scientific proof for the utilization of sesamol, it does not exactly reflect the states in human. In future study, we will perform experiments through the human skeletal muscle cell line and ultimately prove the effects of sesamol via conducting clinical trials. Moreover, we will further clarify the potential mechanism of sesamol using the inhibitors of those key molecular targets.

## Conclusions

**Fig. 8:**
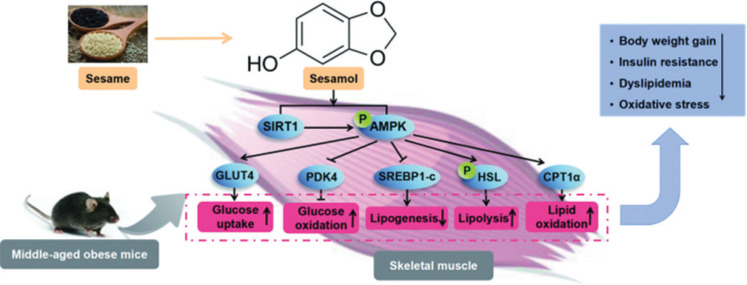
The mechanisms of sesamol on attenuating obesity and its related metabolic syndrome in the middle-aged mice.

It was the first report to indicate that sesamol could alleviate obesity and its related metabolic disorders via regulating glucose and lipid metabolism of skeletal muscle in the middle-aged mice, which might be associated with the stimulation of the SIRT1/AMPK pathway (Fig. 8). The outcomes of the research study provided novel evidences for sesamol as a therapeutic strategy for obesity and its aging associated metabolic syndrome.

## Conflicts of interest and funding

The authors have not received any funding or benefits from industry or elsewhere to conduct this study.

## Author contributions

Conceptualization, M.-M.H. and Q.H.; methodology, J.-H.C.; software, Q.-Q.Z.; validation, M.-M.H. and J.-H.C.; formal analysis, Q.-Q.Z. and Z.-Y.S; investigation, M.-M.H.; resources, Q.H.; data curation, M.-M.H., J.-H.C., Q.-Q.Z., and Z.-Y.S.; writing – original draft preparation, M.-M.H.; writing – review and editing, Q.H. and H.S.; visualization, M.-M.H.; supervision, J.-H.C.; project administration, Q.H.; funding acquisition, Q.H. All authors have read and agreed to the published version of the manuscript.
